# Artificial Intelligence in Cardiac Electrophysiology: A Clinically Oriented Review with Engineering Primers

**DOI:** 10.3390/bioengineering12101102

**Published:** 2025-10-13

**Authors:** Giovanni Canino, Assunta Di Costanzo, Nadia Salerno, Isabella Leo, Mario Cannataro, Pietro Hiram Guzzi, Pierangelo Veltri, Sabato Sorrentino, Salvatore De Rosa, Daniele Torella

**Affiliations:** 1 Department of Experimental and Clinical Medicine, Magna Graecia University, 88100 Catanzaro, Italy; 2 Department of Medical and Surgical Sciences, Magna Graecia University, 88100 Catanzaro, Italy; assuntadicostanzo.med@gmail.com (A.D.C.);; 3 Data Analytics Research Center, Magna Graecia University, 88100 Catanzaro, Italy; 4 DIMES (Dipartimento di Ingegneria Informatica, Modellistica, Elettronica e Sistemistica), University of Calabria, 87036 Rende, Italy

**Keywords:** artificial intelligence, cardiac electrophysiology, deep learning, machine learning, electrocardiogram, atrial fibrillation, ventricular tachycardia, CIED, catheter ablation, wearable devices

## Abstract

Artificial intelligence (AI) is transforming cardiac electrophysiology across the entire care pathway, from arrhythmia detection on 12-lead electrocardiograms (ECGs) and wearables to the guidance of catheter ablation procedures, through to outcome prediction and therapeutic personalization. End-to-end deep learning (DL) models have achieved cardiologist-level performance in rhythm classification and prognostic estimation on standard ECGs, with a reported arrhythmia classification accuracy of ≥95% and an atrial fibrillation detection sensitivity/specificity of ≥96%. The application of AI to wearable devices enables population-scale screening and digital triage pathways. In the electrophysiology (EP) laboratory, AI standardizes the interpretation of intracardiac electrograms (EGMs) and supports target selection, and machine learning (ML)-guided strategies have improved ablation outcomes. In patients with cardiac implantable electronic devices (CIEDs), remote monitoring feeds multiparametric models capable of anticipating heart-failure decompensation and arrhythmic risk. This review outlines the principal modeling paradigms of supervised learning (regression models, support vector machines, neural networks, and random forests) and unsupervised learning (clustering, dimensionality reduction, association rule learning) and examines emerging technologies in electrophysiology (digital twins, physics-informed neural networks, DL for imaging, graph neural networks, and on-device AI). However, major challenges remain for clinical translation, including an external validation rate below 30% and workflow integration below 20%, which represent core obstacles to real-world adoption. A joint clinical engineering roadmap is essential to translate prototypes into reliable, bedside tools.

## 1. Introduction

In recent years, artificial intelligence (AI) has been redefining cardiology, owing to the availability of large datasets derived from high-frequency physiologic signals, multimodal imaging, longitudinal time series, and heterogeneous clinical variables that align well with advances in machine learning (ML) and deep learning (DL) methods [1]. 

**Figure 1:**
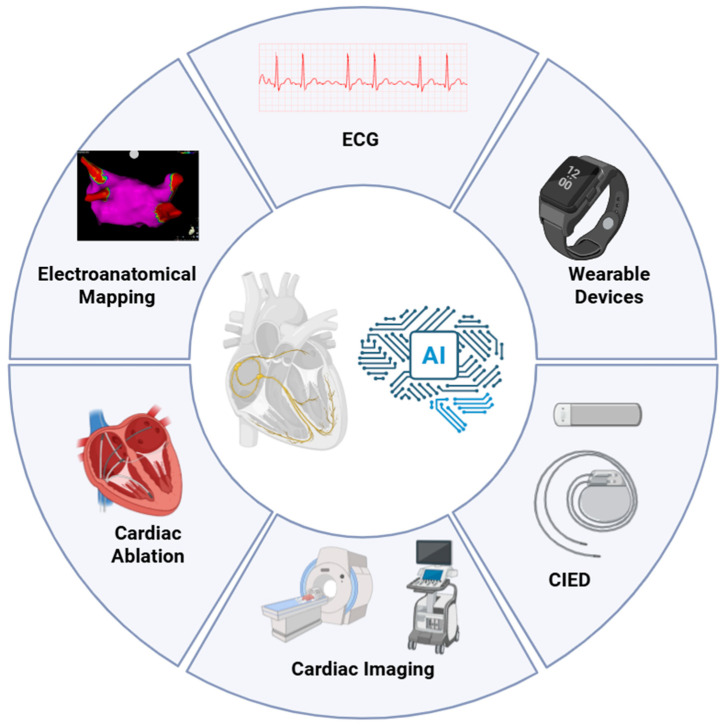
Domains of AI application across the cardiac electrophysiology care pathway. Schematic overview of the principal data sources and procedural touchpoints leveraged by AI in EP. AI integrates non-invasive signals (12-lead ECG and wearable PPG), intracardiac data from the EP lab (EGMs and 3D EAM systems), cardiac imaging (echocardiography, CT, CMR), and continuous device streams from CIEDs to enable screening, risk stratification, guidance of catheter ablation, and longitudinal monitoring. AI, Artificial Intelligence; CIED, Cardiac Implantable Electronic Device; CMR, Cardiovascular Magnetic Resonance; CT, Computed Tomography; ECG, Electrocardiogram.

Specifically, electrophysiology (EP) is the cardiology subspecialty with the broadest availability of structured and semi-structured data, ranging from 12-lead electrocardiograms (ECGs) and photoplethysmograms from wearable devices to intracardiac potentials, three-dimensional (3D) electroanatomical maps (EAMs), and continuous data streams from cardiac implantable electronic devices (CIEDs) [2,3] (Figure 1). 

Within this context, AI has produced particularly robust evidence: automated arrhythmia classification, support for localizing ablation targets, personalized prognostic estimation, and proactive remote monitoring [4]. The recent literature documents an acceleration both in model performance and in the availability of prospective and real-world studies that begin to quantify clinical impact; nonetheless, challenges remain regarding external validation, integration into operational workflows, and model interpretability [5].

On the surface-signal front, the ECG has become the “test bench” of computational cardiology: end-to-end convolutional neural networks (CNNs) trained on raw traces have achieved, or surpassed, cardiologist-level performance in rhythm classification, demonstrating that clinically useful information is encoded in patterns that escape the human eye [6]. The pioneering work by Hannun and colleagues on more than 90,000 ambulatory recordings is often cited as proof of principle for the scalability and accuracy of these approaches [7]. In parallel, the Artificial-Intelligence Electrocardiography (AI-ECG) line of research has expanded the scope from diagnosis to prognosis: algorithms trained on standard ECGs can identify a reduced ejection fraction or occult systolic dysfunction, with prospective validations in routine clinical populations and evaluations in emergency settings [8]. Research has also been facilitated by the availability of large, well-annotated public datasets, such as Public ECG dataset “PTB-XL” (PTB-XL) and its extension PTB-XL+, which enable benchmarking and generalization studies [9,10]. The convergence of AI and wearable devices has extended screening to the population level, opening avenues for digital triage pathways and targeted follow-up (FU) [11].

In patients with CIEDs, remote monitoring enables multiparametric models capable of anticipating heart-failure decompensation and arrhythmic risk [12]. Growing evidence from both prospective studies and real-world data shows that structured alerts embedded within codified care pathways are associated with appropriate management and improved outcomes, offering windows for intervention before clinical deterioration [13].

Concurrently, applications based on DL are increasingly used to connect anatomy and signals: from U-Net architectures for atrial segmentation on late gadolinium enhancement cardiac magnetic resonance (LGE-CMR) or computed tomography (CT) to aid ablation planning to models combining imaging and clinical data to predict responses to cardiac resynchronization therapy (CRT) or post-ablation outcomes [12,14,15,16].

In the EP laboratory, AI supports the standardized interpretation of EGMs and selection of activation patterns that guide mapping and ablation [17,18]. AI models have demonstrated reproducible identification of critical areas in atrial fibrillation (AF) and the classification of arrhythmogenic potentials in ventricular tachycardias (VTs), with concrete promise to reduce inter-operator variability, harmonize substrate tagging, and expedite intraprocedural decision-making [19,20].

A complementary and innovative thread is represented by emerging technologies that integrate electrical signals, geometry, and physics patient specific digital twins (DTs), physics-informed neural networks (PINNs), DL for imaging, graph-based AI on sparse data, and wearables with on-device AI [21,22,23]. These tools enable truly personalized care, reducing procedural time and risk and standardizing workflows without replacing clinical judgment.

The proper application of AI in electrophysiology requires a clear understanding of how models are built, their assumptions, and their limitations. In other words, clinicians and engineers need a common framework and terminology for understanding how models learn, are evaluated, and are validated and monitored over time. Current guidelines reflect this need; they specify methodological and reporting requirements to ensure that AI-based predictive models are developed, tested, and described transparently, avoiding overfitting and systematic bias [24]. 

AI in EP has moved beyond the pioneering phase across multiple domains, from AI-ECG to wearable-based screening and from support for intracavitary mapping to remote monitoring with CIEDs [24]. To translate these innovations into clinical benefit, an integrated clinical engineering workflow is essential, articulating model assumptions, quantifying uncertainty, ensuring calibration and external validation, and designing explainable, physiologically coherent tools [25]. From this perspective, tight integration among data, algorithms, and clinical practice is not optional but foundational to the era of precision medicine, enabling AI to inform truly personalized, timely, and measurable decision-making in electrophysiology.

## 2. Clinical Landscape of Artificial Intelligence in Electrophysiology

**Figure 2:**
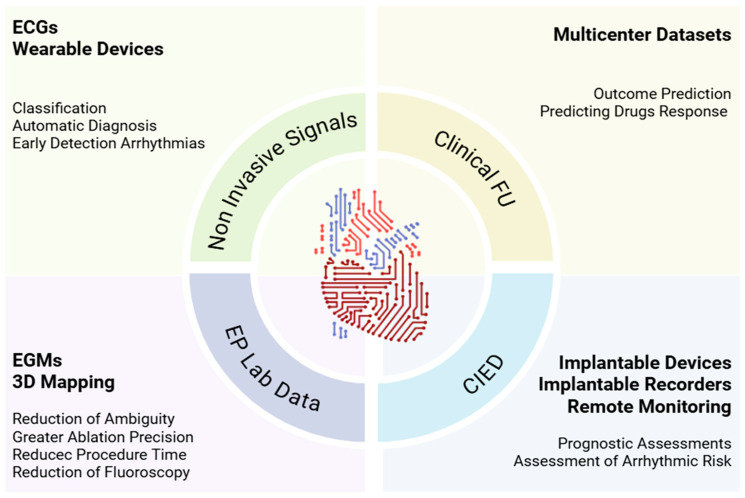
Clinical landscape of AI in EP. AI supports EP from ECGs and wearables that enable earlier arrhythmia detection, to EP-lab learning from EGMs and 3D maps that sharpen ablation and reduce time and fluoroscopy, to CIED-based remote monitoring that refines prognosis and risk. Combined with clinical FU and multicenter datasets, models predict outcomes and drug response, turning routine data into decision support. 3D, Three Dimensional; AI, Artificial Intelligence; CIED, Cardiac Implantable Electronic Device; EP, Electrophysiology; ECGs, Electrocardiograms; EGMs, Intracardiac Electrograms; FU, Follow-Up; Lab, Laboratory.

Clinical electrophysiology generates large volumes of data ideally suited for AI analysis [24]. Applications range from arrhythmia recognition to the guidance of ablation and from outcome prediction to personalized therapy. A useful synthesis distinguishes three principal domains: non-invasive electrocardiographic signals, EP laboratory data (EGMs, EAM, procedural imaging), and longitudinal information from clinical FU and implantable devices (CIEDs) (Figure 2).

Across all three, model performance is rapidly improving, although challenges persist in external validation, workflow integration, and interpretability [5].

### 2.1. Non-Invasive Electrocardiographic Signals

Non-invasive electrocardiographic signals provide an enormous volume of data. Automated ECG analysis was among the earliest applications of AI in cardiology [7]. Standard ECGs produce simultaneous multilead time series from which algorithms can extract beat-to-beat rhythm and P/QRS/ST-T morphology features [26]. Twelve-lead recordings project the same cardiac electrical vector into space, enabling the encoding of conduction blocks, arrhythmias, hypertrophy, and ischemia through inter-lead correlations. Adding clinical metadata (e.g., sex, age, symptoms) further enhances risk stratification [27]. The availability of large public datasets has accelerated research and enabled standardized benchmarking [28]. PTB-XL provides >21,000 recordings and is now a reference dataset for the training/validation of 12-lead ECG algorithms; extensions such as PTB-XL+ add derived features, facilitating studies on explainability and generalization [9].

In this setting, end-to-end DL models, particularly CNNs that apply learnable filters directly to raw traces to autonomously derive discriminative representations, have outperformed handcrafted-feature approaches for tasks such as arrhythmia classification and the detection of latent information not visible to human readers. Hannun and colleagues developed a CNN capable of classifying 12 rhythms from >90,000 ambulatory recordings on single-lead data [7]. These CNNs achieved a mean AUC of 0.97 and an F1 score of 0.837, with “cardiologist-level” performance for automated rhythm classification. Ribeiro et al., in contrast, operated on 12-lead ECGs and targeted six abnormalities (rhythmic and morphological) trained on >2 million exams; on a cardiologist-annotated test set, it reported F1 > 80% for all classes and a specificity of >99% [28]. AI-ECG by Attia et al. is different again: it does not classify rhythm types but detects a latent AF “signature” in sinus rhythm using 649,931 ECGs, with an AUC of 0.87 and an accuracy of ~79–83% [29].

Beyond diagnosis, AI-ECG has shifted the focus toward prognosis: algorithms trained on standard ECGs can identify reduced left-ventricular ejection fraction in prospectively validated settings, anticipate the need for echocardiography, and flag at-risk individuals even in the absence of evident clinical signs [30]. For arrhythmias, DL models demonstrate sensitivity/specificity comparable to, or exceeding, traditional methods for AF detection, including challenging contexts such as paroxysmal episodes and noisy signals [29].

In parallel, the advent of wearable devices (e.g., smartwatches) has opened the door to continuous, population-level screening, enabling the earlier identification of clinically silent arrhythmias. Clinically approved (FDA/CE) applications include on-device single-lead ECGs (e.g., smartwatch iECG, handheld devices/ECG patches) and large-scale PPG screening modules [31]. For iECG devices, the most informative metrics are sensitivity/specificity and, for notification algorithms, positive predictive value, along with practical indicators such as signal quality and the proportion of inconclusive tracings [23,32,33,34,35,36]. The Apple Heart Study demonstrated the feasibility of AF detection via photoplethysmography (PPG) in very large cohorts, inaugurating a new era of digital cardiology [37]. For clinical ECG patches, studies more often report diagnostic yield relative to the 24 h Holter rather than per-patient sensitivity/specificity, reflecting their role in detecting paroxysmal arrhythmias over prolonged monitoring windows (Table 1) [35,38].

Beyond marketed devices, a clinical-validation tier includes AI-ECG models that, while not yet cleared for specific diagnostic indications, show prospective and implementation-level performance. Examples are algorithms that infer an “AF signature” in sinus rhythm to flag individuals at risk and extended-wear strategies embedded in care pathways that improve diagnostic efficiency and may accelerate anticoagulation decisions [39]. 

At the laboratory/early-stage tier, methods remain preclinical but promising. Graph neural networks can prioritize isthmus regions in scar-related atrial tachycardia for targeting, while other deep-learning approaches refine AF risk estimation or mapping from limited inputs [40]. These require multicenter prospective validation and the assessment of clinical impact before translation. 

In summary, the ECG is an ideal substrate for supervised end-to-end models owing to the richness of its time series, the relative reliability of labeling, and the availability of large datasets.

### 2.2. Electrophysiology Laboratory Data

During invasive procedures, the large amount of data collected via multipolar catheters (high-density EGMs and EAM) and 3D imaging systems represent a remarkable resource for AI applications. 

Key algorithmic objectives include standardizing and accelerating signal interpretation, identifying informative substrate/ablation targets, and reducing procedural times. Machine learning can standardize electrogram interpretation, reduce ambiguity, and offer a platform potentially usable in real time to support mapping and ablation decisions [41]. For example, to overcome inter-observer variability in EGMs signal analysis during AF, Alhusseini and colleagues showed that a CNN can objectively recognize complex activation patterns on bi-atrial maps and guide AF ablation [42]. Beyond AF, AI has also shown utility in VT for recognizing arrhythmogenic patterns and classifying potential targets. In post-ischemic VT, a supervised classifier trained on EGMs identified arrhythmogenic ventricular potentials, decreasing reliance on purely visual interpretation and standardizing selection of ablation sites [19].

Machine learning is also becoming a practical assistant for determining ablation sites in both AF and VT. In AF, the randomized TAILORED-AF trial showed that an ML-guided strategy—identifying spatiotemporal dispersion areas on EGMs and treating them in addition to pulmonary vein isolation (PVI)—was superior to PVI alone in achieving AF freedom at 12 months (88% vs. 70%), demonstrating that objective, reproducible signal analysis can effectively direct lesion selection [11]. 

In post-ischemic VT, Baldazzi et al. developed and validated a supervised approach to automatically recognize abnormal ventricular potentials on bipolar electrograms, achieving cross-validation accuracies of ≥93% [19]. Model-flagged sites aligned with en-trances/isthmuses and regions ultimately ablated, suggesting the feasibility of standardizing substrate tagging and expediting target identification, particularly in complex circuits. From an efficiency standpoint, Fox et al. reported that AI-assisted, 12-lead, ECG-informed arrhythmia mapping was associated with shorter procedures (−22.6%) and reduced fluoroscopy time (−43.7%) without adverse effects on outcomes or complications; 6-month arrhythmia-free survival was 73.5% (63.3% in the control group) [43].

### 2.3. Longitudinal Information from Clinical Follow-Up and Implantable Cardiac Electronic Devices (CIEDs)

Another rapidly expanding area is outcome prediction after pharmacologic therapy or ablation. Supervised algorithms trained on large multicenter datasets have shown the ability to integrate clinical, electrophysiological, and imaging variables to estimate individual risk, an approach aligned with personalized medicine that aims to prospectively identify patients most likely to benefit from a given treatment. In the cardioversion setting, ML models combining clinical features and ECG have accurately predicted short-term recurrence and suggested personalized surveillance schemes [44,45]. In parallel, exploratory in silico studies have demonstrated the feasibility of predicting antiarrhythmic drug responses in virtual populations, which is useful for hypothesis generation and rhythm-control selection [46].

In recent years, supervised models have achieved good accuracy in predicting AF recurrence after ablation by combining clinical, electrocardiographic, and imaging variables [47,48,49,50].

Extending these predictive frameworks from static, episode-based datasets to continuous, real-world physiology, CIEDs and implantable loop recorders provide continuous data streams and a privileged substrate for predictive models, also thanks to remote monitoring. The most robust evidence concerns the prediction of heart failure (HF) decompensation and arrhythmic risk (Table 2). In patients monitored remotely, multiparametric algorithms trained and validated in prospective cohorts are automating HF risk estimation. Prospective and real-world studies are consolidating the association of structured alerts—embedded in codified care pathways—with improved outcomes and reduced rehospitalizations when integrated into organized clinical workflows [42,51,52,53,54]. On the arrhythmic front, daily analysis of remote Implantable Cardioverter-Defibrillator (ICD) data enables models that predict imminent appropriate therapies, electrical storms, or AF recurrence, offering a window to optimize medical therapy, device programming, or the timing of interventions [55,56].

The integration of AI into clinical decision support systems (CDSS) for electrophysiology is consolidating. The goal is not to replace clinicians but to standardize high-variability steps and accelerate repetitive decisions from signals and maps.

In summary, the clinical landscape indicates that AI has already produced tools with potentially meaningful impact in everyday practice. To make this impact systematic, mechanisms must be made explicit, training/validation criteria transparent, and the limitations hampering adoption acknowledged. Here, the engineering contribution is decisive: translating algorithms into robust, explainable, interoperable solutions; ensuring calibration, safety, and post-deployment monitoring; and enabling the prospective evaluation of clinically relevant endpoints. 

## 3. Technical Foundations and Engineering Considerations

In cardiac electrophysiology, AI plays a pivotal role in advancing new diagnostic and therapeutic techniques. Machine learning (ML) is an active research area with broad clinical application [57]. Understanding the underlying models and learning principles is essential to assess the utility, reliability, and limitations of AI in electrophysiology. The ML methods most frequently applied in this field are supervised (Table 3) and unsupervised learning (Table 4).

### 3.1. Supervised Learning

Supervised algorithms are widely used and are trained on datasets containing both input data “X” (ECGs, EGMs, clinical data, imaging data, procedural parameters) and the correct outputs “y” (diagnosis, site of origin, risk of recurrence, time-to-event). The model learns the mapping from inputs to the correct outputs (X→y) with the goal of generalizing to unseen data and answering the relevant clinical question. Here, labeled input are needed; the latter are generally the responsibility of a human operator to ensure the truthfulness and correctness of the information.

#### 3.1.1. Least-Squares Regression (LSR)

Least-squares regression (LSR) characterizes the relationship between a continuous variable “y” (ms, mV, Ω, mm, %) and one or more measured inputs. LSR estimates the line that passes “as close as possible” to all points by minimizing the sum of squared residuals (vertical point-to-line distances), yielding a rule for predicting values at new points and coefficients (Δ) that quantify each variable’s contribution to the output. When relationships are nonlinear (e.g., exponential or logistic), LSR can be extended to fit curves rather than straight lines. 

In electrophysiology, LSR is applied according to the clinical question, classification, regression, survival/time-to-event, or segmentation. Classification is used to categorize inputs; for example, ML models have classified the site of origin of VT from the ECG [58]. Regression is used when the outcome is continuous, such as estimating an index, burden (%), conduction velocity (CV), local impedance, or lesion depth from procedural parameters like power, duration, contact force, or impedance. Sprenger et al. used regression to predict the Ablation Index (AIx) and Lesion Size Index (LSI) during radiofrequency (RF) ablation for AF from local impedance values and application duration [59]. Giffard-Roisin et al. applied a regression model to predict response to CRT using non invasive data; body-surface potential mapping enabled the preoperative estimation of activation onset and tissue conductivity [60]. For survival outcomes, the objective is time-to-event, widely used to predict time to recurrence after AF ablation [61,62,63]. Segmentation models provide “point-by-point” maps, classifying each site as healthy, border, or fibrotic based on intracardiac signals and enabling identification of target zones for ablation [64].

#### 3.1.2. Support Vector Machines (SVMs)

Support vector machines (SVMs) are fast and efficient models, particularly useful in electrophysiology when datasets are relatively small but high-dimensional. Their primary function is classification by identifying a decision boundary (hyperplane) that separates classes. In simple cases, the boundary is linear; for nonlinearly separable data, a curved hyperplane in a transformed feature space is used. 

In electrophysiology, SVMs have been applied to AF screening from ECG, risk stratification, and prognostic prediction from electrophysiological signals [65,66,67,68]. For screening, a major application is arrhythmia detection from short, single-lead ECGs recorded by wearable devices [69,70,71,72]. For sudden cardiac death risk, Rodriguez et al. used an SVM model in dilated cardiomyopathy based on heart-rate variability (HRV) and blood-pressure variability (BPV) [73]. SVMs have also been used to estimate postoperative AF risk from long-term ECG analysis and to predict favorable prognosis (CRT response) or adverse outcomes as VT or ventricular fibrillation (VF) susceptibility in ischemic cardiomyopathy [67,74,75].

#### 3.1.3. Supervised Neural Networks

Supervised neural networks (NNs) comprise “artificial neurons” arranged in layers that receive inputs, process them, and produce outputs. During training, networks are provided both raw inputs and the corresponding correct outputs, learning complex patterns that elude traditional models.

Clinically, NNs are widely used because they can learn the relative importance of features and infer relationships to generate predictions such as AF onset, HF progression, post-MI arrhythmia, or diagnoses of conditions predisposing to sudden cardiac death. Several studies, including the algorithm by Attia and colleagues, trained networks to diagnose AF from ECGs recorded during normal sinus rhythm [76,77]. Another application is the early detection of rare cardiac diseases strongly associated with sudden death; for example, Goto et al. developed a model for ECG-based prescreening of cardiac amyloidosis [78]. Augusto et al. reported the superior, reproducible automated measurement of maximal left-ventricular-wall thickness, with implications for the timely diagnosis and treatment of hypertrophic cardiomyopathy. Prognostic modelling is also feasible: MacGregor et al. built an algorithm to identify patients with dilated cardiomyopathy likely to respond to medical therapy alone [79]. Multiple NN models have been developed to stratify arrhythmic risk in post-MI patients [80,81].

#### 3.1.4. Random Forest

Random forests consist of ensembles of decision trees built via bootstrapping, with each tree trained on heterogeneous samples from the same dataset (clinical features, derived ECG/EGM variables, imaging) and random feature subsets at each split. For classification, predictions are made by majority vote (or averaged probabilities); for regression, they are made by averaging tree estimates. 

This approach is widely used to predict responses to CRT using multiple features, including clinical data, ECG variables, echocardiographic measurements, biomarker levels, and left-ventricular lead position [82,83]. Random forests have also been used to estimate recurrence risk after ablation and are commonly applied in screening to identify features associated with AF diagnosis [84,85,86].

### 3.2. Unsupervised Learning

Unlike supervised learning, where algorithms learn from labelled data (inputs with corresponding correct outputs), unsupervised learning relies on unannotated datasets. The goal is not to “predict” a known outcome but to identify hidden structures, relationships, patterns, or clusters. This is especially promising in electrophysiology, where signals are often complex, noisy, and non-intuitive, and where a diagnostic gold standard or well-defined outcomes may be lacking. Unsupervised algorithms can generate new clinical hypotheses, explore patient/signal subgroups with shared characteristics, and uncover recurrent electrophysiological patterns that guide therapy personalization or reveal new ablation targets.

#### 3.2.1. Clustering

Clustering is the most common technique for automatically grouping data into homogeneous clusters. K-means clustering partitions data into k groups by minimizing distances to cluster centroids [87]. DBSCAN (Density-Based Spatial Clustering of Applications with Noise) is advantageous for irregular distributions, identifying “dense” regions and separating them from sparse, noisy areas [88]. Hierarchical clustering constructs a dendrogram to explore cluster structure at different levels of granularity [87].

In electrophysiology, clustering is mainly used to phenotype patients or signals into homogeneous groups. Zhang et al. stratified participants in the CABANA trial, identifying AF phenotypes with distinct clinical and prognostic features [89]. Treatment personalization, particularly risk categorization, has become a key objective of contemporary clinical studies. Clustering has also been applied to distinguish normal potentials, fractionated signals, and low-voltage activity during EAM, improving identification of critical zones (e.g., macro-reentry pathways or abnormal conduction) for tachyarrhythmia ablation [90]. Kong et al. used clustering to select the optimal filter to remove loss-of-contact and noisy-electrode artifacts when identifying active sites [91].

#### 3.2.2. Dimensionality Reduction

Electrophysiology datasets often comprise thousands of variables (ECG features, imaging parameters, clinical variables, intracardiac signals), necessitating dimensionality reduction. This is a crucial analytical step: it improves visualization, reduces noise, supports clustering, and facilitates more robust models. 

Principal component analysis (PCA) identifies principal components—new variables formed as linear combinations of the originals—that explain most of the dataset variance. Dimensionality reduction is frequently used as a preprocessing step for other algorithms, removing redundancy and enhancing interpretability. Yang et al. highlighted PCA use in ECG processing for dimensionality reduction and feature extraction, confirming associations between specific features and future AF onset [92].

Newer methods such as t-Distributed Stochastic Neighbor Embedding (t-SNE) and Uniform Manifold Approximation and Projection (UMAP) provide intuitive two-dimensional representations that preserve local similarity structure. Sánchez Carballo et al. showed that UMAP can reduce high-dimensional ECG data into a more interpretable 2D “map” [93]. Signals from anatomically adjacent cardiac regions clustered together in this 2D space, suggesting that UMAP can yield a “simplified electrical map” that captures local electrical patterns without the full complexity of the original signals.

#### 3.2.3. Association Rule Learning

Association rule learning (ARL) identifies frequent co-occurrences among clinical events or features. 

In electrophysiology, ARL can reveal recurrent patterns linking clinical characteristics and arrhythmia types, or procedural parameters and ablation failures [94]. Direct applications in EP remain sparse but represent a promising area [95]. 

#### 3.2.4. Unsupervised Deep Learning

DL has transformed signal and image analysis by enabling CNNs to automatically extract salient features from complex data such as 12-lead ECGs or cardiac MRI images. The main limitation remains the opacity of decision processes, the “black box”, raising concerns about trust and interpretability in clinical settings [96]. 

From an engineering standpoint, there is growing interest in explainable AI (XAI), designed to deliver not only predictions but also insight into the reasoning behind them [97,98]. In electrophysiology, this may include highlighting ECG segments most responsible for a tachycardia classification. Such transparency is essential to foster clinician acceptance.

In summary, the engineering foundations provide clinicians with the vocabulary and conceptual framework needed to appraise the reliability of AI models and to engage meaningfully with developers in translating algorithms into robust, explainable, and interoperable tools for clinical electrophysiology.

## 4. Emerging Technologies

Alongside established applications, several technologies have recently emerged that, through integration with AI, promise to transform clinical electrophysiology (Table 5).

In the era of precision medicine, tailoring therapies to the individual patient has become a central objective of care [99].

### 4.1. Digital Twin

A DT is a patient-specific model that replicates cardiac anatomy and physiology, enabling the simulation of electrical activity and in silico testing of therapeutic strategies before intervention [100,101,102]. 

In AF, patient-specific “virtual ablation” on reconstructed atrial geometries has allowed for the comparison of lesion strategies and was followed by a multicenter randomized study demonstrating feasibility and the ability to predict the most effective lesion sets in persistent AF [103]. Haïssaguerre and colleagues combined high-density ECG with chest CT to reconstruct bi-atrial activation patterns in real time and identify AF drivers (foci/rotors) [104]. 

For VT, personalized “virtual-heart” models derived from LGE-MRI/CT can predict minimal ablation targets and guide procedures even without invasive mapping [105]. A complementary approach is the reentry vulnerability index (RVI): simple pacing protocols yield maps comparing local recovery (repolarization) with activation time between neighboring points, highlighting regions most susceptible to reentry [106]. In practice, low-RVI clusters co-localize with exits/isthmuses of scar-related VT and indicate effective ablation sites. Additional tools, such as the integration of CT-derived intramyocardial/epicardial fat marker (inFAT), further refine target selection [107]. 

In cardiac pacing, a major challenge is predicting responses to CRT, given the proportion of non-responders. Mechanical digital-twin “virtual pacing,” personalized using strain and echocardiography, has predicted left-ventricular reverse remodeling after CRT, suggesting a role for DT in candidate selection and therapy optimization [108]. A patient-specific electromechanical model showed that length–tension dependence (Frank–Starling) can already synchronize stress/strain; in such cases, the incremental benefit of CRT may be limited [109]. To support candidate selection, Lumens et al. proposed the Systolic Stretch Index (SSI), which distinguishes true electromechanical discoordination from hypocontractility/scar and predicts post-CRT outcomes [110].

Operationalizing the twin paradigm requires open tools: platforms such as pyCEPS convert proprietary EAM data into open formats, facilitating calibration, integration, and reproducibility [111]. However, use is currently limited to a few major mapping systems (CARTO 3 and EnSite Precision); conversion is not yet universal, and centers using unsupported systems may experience lower conversion success rates. The openCARP v18.1 ecosystem provides an open, reproducible environment (with Python 3.10.12 pipelines) that promotes standardization, transparency, and shareable simulation workflows—key prerequisites for robust, AI-integrated DT [112]. However, simulations with realistic anatomy and high-resolution meshes can take hours for a single-patient model, which does not meet intraoperative real-time needs; current practice is preoperative use with offline calibration and simulation.

A recent review proposed a unifying framework for electrophysiology DT, emphasizing the transition from static models to continuously updated, highly calibrated, patient-specific twins that seamlessly integrate imaging, signals, and clinical data [113]. Looking ahead, DTs may become operational tools to guide personalized decisions, shorten procedures, and increase ablation safety. In summary, preoperative digital-twin simulations can streamline ablation—reducing the number of lesions/passes and enabling more targeted substrate selection—and limit iterations during mapping. These benefits, however, require upfront investments (engineering time, high-resolution imaging/segmentation, computation) and dedicated infrastructure (servers, storage, quality assurance). To date, peer-reviewed, longitudinal cost-effectiveness evidence specifically for digital twins in electrophysiology remains limited.

### 4.2. Physics-Informed Neural Networks

PINNs are hybrid learning approaches in which neural networks are trained not only on data but also on the physical equations governing myocardial electrical conduction [114]. 

In practice, PINNs do not just learn from data; they embed physical laws into training. The loss function combines a data term and a physics term (equation residuals), so the model is penalized both when it deviates from measurements and when it violates physiology. Key laws/constraints in cardiac electrophysiology:

- Eikonal equation (activation times). Guides reconstruction of activation-time maps: where conduction is slow (scar/fibrosis), activation must occur later. The PINN prevents unrealistic “jumps” of the wavefront.
- Monodomain/bidomain models (current propagation). Describe how current spreads in an anisotropic myocardium (easier along fibers, harder across) and how it depends on ionic currents (e.g., Hodgkin–Huxley, ten Tusscher–Panfilov, O’Hara–Rudy). The PINN enforces charge conservation, anisotropy, and consistency with ionic models, reducing non-physiologic solutions.
- Boundary/initial conditions. At tissue borders (e.g., chambers or non-conductive scar) current does not cross the boundary (no-flux/Neumann). The PINN respects these anatomical “walls”.
- Physiological constraints. Clinical guardrails: conduction velocity ≥ 0, diffusivity ≥ 0, APD within plausible ranges, anisotropy aligned with fiber orientation. The PINN penalizes solutions outside these ranges.

In electrophysiology, they enable the reconstruction of activation maps from sparse, noisy measurements; the estimation of tissue parameters (diffusivity/CV, anisotropy, excitability and recovery properties); and solutions to inverse problems such as electrocardiographic imaging (ECGi), which infers cardiac electrical activity from torso-surface signals [22]. Compared with purely data-driven methods, PINNs are more robust and yield results that are physiologically plausible and interpretable. 

Sahli-Costabal et al. used a PINN constrained by the eikonal equation to produce realistic activation and conduction-velocity maps from few measurements; an active-learning component that recommends the next sampling points suggests the potential to reduce procedural time and improve mapping accuracy [115]. In parallel, Grandits et al. applied PINNs to identify tissue properties (anisotropy, fiber orientation, conductivities) directly from EAM [116]. The concept was extended with FiberNet, which integrates multiple activation maps to robustly infer local fiber orientation, moving closer to truly patient-specific models [117]. Herrero Martin et al. demonstrated tissue characterization and therapy guidance using PINNs: from sparse, noisy data, they reconstructed the full action potential, estimated electrophysiological parameters (Action Potential Duration or APD, excitability, diffusion), and assessed sensitivity to antiarrhythmic drug effects [22]. Dermul et al. unified mechanical and electrical signals with PINNs, reconstructing activation from tissue motion and opening avenues for non-invasive mapping with potential reductions in procedural time and risk [118].

ECGi is a non-invasive technique that, using a multielectrode vest and chest/heart CT or MRI, reconstructs epicardial potentials and activation times via inverse algorithms [119]. In ECGi, Bacoyannis et al. proposed a generative deep-learning approach (Conditional Variational Autoencoder or CVAE) that integrates anatomy and torso potentials to estimate volumetric activation maps probabilistically, showing that data-driven models can capture the spatiotemporal correlations of the inverse problem [2]. More recently, Zhu et al. introduced a PINN with residual learning and local spatiotemporal support that mitigates typical PINN limitations (overfitting to collocation points, training instability, poor scalability), achieving more accurate, noise-robust ECGi reconstructions [120]. Collectively, these studies advance hybrid models in which data and physics combine to improve non-invasive inversion of cardiac electrical activity [120]. 

Overall, PINNs offer a promising bridge between biophysical simulation and AI, enabling clinically usable cardiac DTs.

### 4.3. Deep Learning for Cardiac Imaging

DL is already widely used in cardiovascular imaging, and EP-focused applications are expanding rapidly. The automated segmentation of atrial and ventricular chambers enables the rapid, precise reconstruction of structures for use in mapping systems. 

U-Net–type architectures segment the left atrium (LA) and myocardium on LGE-CMR, reducing time and inter-operator variability and enabling standardized workflows for ablation planning and the construction of patient-specific models [15,121,122]. Applying U-Nets to cardiac CT similarly segments the LA/right atrium (RA) and epicardial adipose tissue (EAT) within seconds to quantify remodeling and predict AF recurrence after ablation [14]. On intracardiac echocardiography (ICE), dedicated algorithms guide view selection and are improving the robustness of 3D left-atrial rendering, reducing operator dependence [123,124]. Beyond segmentation, DL is informing clinical decision-making: models that integrate imaging (CT/LGE/echo) with clinical data predict AF ablation outcomes and CRT response [48,116,125]. For electro-functional imaging, DL-based ECGi seeks to translate surface ECGs into 3D activation maps, fusing anatomical images (CT/CMR) with electrical signals to non-invasively reconstruct cardiac activity [2]. 

In summary, DL applied to LGE-CMR, CT, and echocardiography provides complementary building blocks, segmentation, fibrosis/remodeling quantification, and predictive modeling, which accelerate data-driven EP workflows and the transition to patient-specific models integrable with mapping systems [126].

### 4.4. Graph-Based AI and Convolutional Models for Sparse Data

A rapidly growing area is the use of graph convolutional neural networks (GCNNs) to extract clinically useful information from EGMs acquired with multipolar catheters. These algorithms handle unstructured, incomplete datasets and can estimate complex electrophysiologic parameters even when mapping points are irregularly distributed, raising the possibility of shorter procedures without loss of accuracy. 

In the atria, GCNN trained on EAM have predicted critical isthmuses in scar-related tachycardias, showing that learning on graphs can identify ablation targets from imperfect point sets [40,127]. In the ventricles, a GCNN trained on realistic simulations and integrated with 12-lead ECG and CMR reconstructs 3D activation-time maps from sparse points and can suggest subsequent sampling locations, with the potential to reduce procedural duration [128]. 

For ECGi, recent studies indicate that GNNs may reduce the number of electrodes while preserving reconstruction quality—a step toward less-burdensome, faster-to-configure systems [129]. 

This line of work complements other DL approaches operating on intracardiac signals and maps, reinforcing the concept that geometry-aware models can accelerate mapping, decrease dependence on sampling density, and standardize intraprocedural interpretation.

### 4.5. Advanced Wearables and On-Device AI

Prevention and monitoring are increasingly centered on wearable devices, enabling large-scale screening and timelier arrhythmia management; recent reviews of AI-enabled wearable ECG confirm the trend and growing clinical impact in electrophysiology [130]. Hardware–software advances are moving computation onto the device (edge-AI/TinyML), allowing for continuous signal analysis and real-time notifications [131]. From a regulatory perspective, on-device features (Apple and Samsung Irregular Rhythm Notification, Apple AFib History, Fitbit AF algorithms) are already marketed as screening support tools [132,133].

Diagnostic performance is high for AF screening with smartwatches, Holters, and ECG patches (sensitivities/specificities ~96–98%), particularly for event detection in post-cryptogenic-stroke screening [134,135]. Novel form factors are emerging: smart rings have early validations for AF and, experimentally, for ventricular arrhythmias [136,137]; in parallel, e-textiles and multichannel wearables show Holter-comparable accuracy in daily practice with greater comfort and adherence [138].

Overall, wearables are evolving from simple sensors to decision-support tools that can be integrated into EP workflows, from remote triage to FU and procedural planning, while generating standardized datasets for patient-specific modeling [139].

In summary, these emerging technologies do not replace traditional clinical approaches; rather, they augment them, providing tools that may reshape the practice of electrophysiology in the coming years.

## 5. Ethical, Legal, and Regulatory Considerations for AI in Cardiac Electrophysiology

Despite the impressive achievements of artificial intelligence (AI) in cardiac electrophysiology (EP), several limitations and challenges must be critically acknowledged before large-scale clinical implementation.

### 5.1. Ethical Considerations

AI can broaden access, standardize decisions, and anticipate risk windows at low marginal cost. However, these advantages hinge on the quality and representativeness of the data. Biases in training datasets can produce systematic disparities in diagnostic accuracy across gender, ethnicity, or device type [140,141,142]. Mitigation requires the deliberate inclusion of underrepresented populations, periodic auditing, and the use of explainable AI (XAI) methods [7,143]. Patient trust is fundamental: patients should be aware that AI contributes to, rather than replaces, clinical decision-making [144]. Transparent XAI outputs and traceable algorithmic reasoning are crucial to address “black-box” concerns and to sustain clinician acceptance [143,145]. Finally, the introduction of AI-based decision support should not widen health inequities between well-resourced centers and facilities with limited resources [146].

### 5.2. Regulatory and Legislative Challenges

In the United States, AI-enabled medical devices are reviewed by the FDA via one of three premarket pathways: 510(k) clearance, De Novo classification, or Premarket Approval (PMA). As most AI software qualifies as Software as a Medical Device (SaMD), development should align with Good Machine Learning Practice (GMLP) principles and, where applicable, a Predetermined Change Control Plan (PCCP). GMLP provides ten lifecycle-wide principles, from design and data governance to clinical validation and post-market monitoring, that function as a practical checklist for submissions and audits. For models that learn or update over time, the PCCP final guidance allows manufacturers to predefine which elements of the algorithm may evolve, how those changes will be controlled, and which guardrails/metrics will be tracked post-market, enabling iterative updates without repeating a full authorization each time.

In the European Union, Regulation (EU) 2017/745 (MDR) strengthens safety, performance, and post-market surveillance requirements for CE marking [147]. The Medical Device Coordination Group (MDCG) 2019-11 Rev.1 remains the key guidance for software qualification/classification. The EU AI Act adds AI-specific, horizontal obligations (risk management, data governance, transparency, logging, human oversight) on top of MDR. In parallel, General Data Protection Regulation (GDPR) for EU and Health Insurance Portability and Accountability Act (HIPAA) for US require robust data protection and cybersecurity [148,149,150]. 

The spread of AI-enabled wearables for arrhythmia detection has raised data-privacy concerns. PPG/ECG signals are personal health data. Recent analyses indicate that only about one-third (~30–34%) of manufacturers implement encryption and key management in line with protection standards across the entire data pathway, creating a risk of information leakage [151]. On-device AI reduces exposure but does not eliminate security and privacy issues [152]. Accordingly, data minimization, Data Protection Impact Assessments (DPIAs), independent audits, and traceable logging are needed. In addition, there is a lack of specific regulatory guidance for AI algorithms that process cardiac rhythm data in minors (covering consent, parental access, retention, and profiling), warranting pediatric-specific safeguards [153].

Medico-legal liability for algorithmic error remains a legal gray area, underscoring the need for explicit human oversight, clear accountability, and auditable versioning/logging throughout the AI lifecycle [154].

### 5.3. Clinical Validation and Generalizability

AI has considerable potential in cardiology, yet many models still lack robust external validation on independent cohorts, limiting their reliability beyond the development setting [25]. Fewer than one-third of cardiology AI studies undergo external validation, undermining real-world clinical utility [155]. Real-world heterogeneity, data drift, and differences in acquisition hardware can further erode performance, making in-production monitoring and model maintenance plans essential [156,157]. To ensure both generalizability and clinical benefit, multicenter, prospective studies with clinically meaningful endpoints are needed, ideally designed and reported according to SPIRIT-AI and CONSORT-AI extensions for AI-enabled clinical trials [144,158].

### 5.4. Integration and Application

For benefits to translate meaningfully to the bedside, healthcare systems need interoperability with electronic health records and mapping platforms, continuous MLOps-based model monitoring, and comprehensive staff training [159]. A responsible AI framework, transparent, explainable, fair, and subject to ongoing validation, is essential for clinical acceptance [24,160]. Multidisciplinary collaboration among clinicians, engineers, bioethicists, and regulators should guide the entire AI lifecycle, ensuring that innovation yields a safer and more equitable electrophysiology practice. Organizational readiness, workflow redesign, role definition, training, and sustainable maintenance costs, often determines success or failure [159,161]. When these conditions are met, evidence points to system-level gains: broader and more sustainable screening, more reproducible in-lab decision-making, earlier interventions, and more personalized therapies.

## 6. Conclusions

Cardiac electrophysiology is entering a phase in which AI is no longer merely a technical accessory but a structural component of diagnostic, therapeutic, and FU pathways.

Evidence accrued across ECG, wearable devices, intracardiac mapping, imaging, and remote monitoring demonstrates concrete clinical value, standardizing decisions, anticipating windows of risk, and personalizing procedural choices. The most robust signal comes from AI-ECG, where end-to-end NNs have achieved cardiologist-level performance for rhythm classification and, crucially, have shifted the emphasis toward prognosis by identifying latent risk phenotypes from standard traces [7,8,9]. Expansion to wearables has enabled population-scale screening and digital triage pathways driven by algorithmic alerts, with feasibility demonstrated in very large cohorts [37]. In the EP laboratory, supervised models applied to EGMs recognize complex activation patterns, reduce inter-operator variability, and assist target selection in AF and VT; early clinical data indicate shorter procedures and reduced fluoroscopy without compromising outcomes [19]. On the FU side, CIEDs have turned telemetric streams into actionable clinical signals: multiparametric scores anticipate heart-failure decompensation and arrhythmic instability, and prospective/real-world studies suggest that, when embedded within structured pathways with dedicated teams, they can reduce rehospitalizations and enable earlier interventions [13,51,53]. 

The technological trajectory further suggests that progress will not derive solely from larger or more efficient models but from integrated systems that fuse signals, images, and the physical laws of cardiac conduction. In this context, patient-specific DTs enable pre-procedural simulation, “virtual ablation” in AF, and the identification of minimal VT targets, with feasibility and predictive performance that foreshadow operational use for the planning and quality control of ablation strategies [103,105,108]. In parallel, PINNs, by embedding conduction physics alongside measured data, offer more robust and interpretable solutions to inverse problems and to tissue-property estimation from sparse measurements; prospectively, they may shorten mapping times and intelligently guide acquisition of additional points, with tangible intra-procedural potential [22]. Imaging powered by DL completes the picture: U-Net–type architectures markedly shorten atrial and myocardial segmentation on LGE-CMR/CT, enhance reproducibility of substrate quantification, and integrate with multimodal models that predict CRT response and post-ablation outcomes, laying the groundwork for truly patient-specific workflows [125,126]. Finally, the adoption of GCNN on electro-anatomic graphs shows that clinically useful information can be extracted even from incomplete maps, opening avenues for greater procedural efficiency [40]. 

Against this backdrop, concrete advantages are emerging. Scalability enables broader screening and risk stratification at low marginal cost per evaluation [7]. Standardization in the EP lab, via algorithmic electrogram analysis, reduces inter-operator variability in activation-pattern interpretation and yields more uniform substrate tagging, directly informing mapping and ablation decisions [11]. Timeliness improves through multiparametric algorithms from remote monitoring, which identify hemodynamic and arrhythmic instability earlier and trigger response pathways before clinical decompensation [162]. Personalization is now tangible through integration of imaging, signals, and simulation [105]. Collectively, these benefits point to potential system-wide impact: more accessible and equitable pathways through scalable screening, more reproducible in-lab decision-making, earlier interventions enabled by telemonitoring, and therapies better tailored to individual patients [11].

Important limitations remain. Generalizability can be undermined by selection bias, distribution shift, and noisy labels; addressing these issues requires multicenter consortia and robustly annotated datasets, together with interoperable tools and formats that promote reproducibility and technology transfer [25,111,112,163,164]. Interpretability must advance toward explanations that are clear, stable, and physiologically coherent [140,141]. Safety demands prospective studies, continuous post-deployment monitoring, and explicit rules for clinician intervention when the model is uncertain or operating out of distribution [143,144,146,165]. Organizational factors ultimately determine bedside value: seamless IT integration, staff training, clearly defined roles, and medico-legal responsibilities across the algorithm’s lifecycle [148,154,156]. Finally, sustainability should be demonstrated in pragmatic trials incorporating rigorous economic analyses and pathway impact, to justify large-scale adoption (Table 6) [159,160,161].

**Figure 3:**
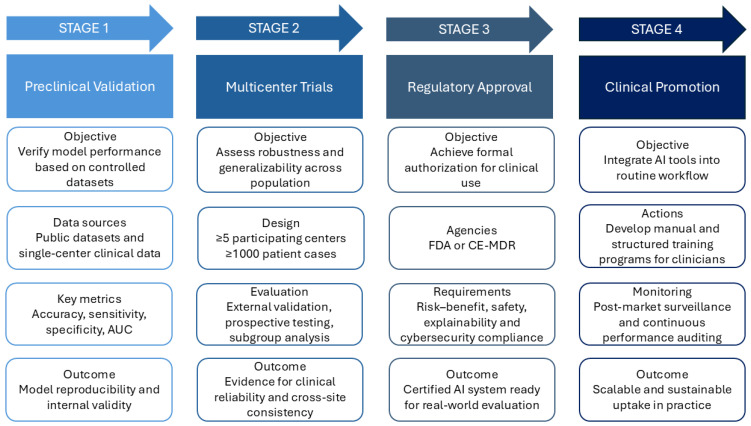
Clinical translation roadmap for AI in cardiac electrophysiology. Four staged pathways from laboratory prototype to routine care. Stage 1—Preclinical Validation: verify model performance on controlled datasets and single-center data. Stage 2—Multicenter Trials: ≥5 centers and ≥1000 cases to assess robustness and generalizability. Stage 3—Regulatory Approval: submissions to FDA or CE-MDR with documented risk–benefit, safety, explainability, and cybersecurity compliance. Stage 4—Clinical Promotion: integrate into routine workflow with an AI operation manual, structured clinician training and monitor via post-market surveillance and continuous performance auditing. AI, Artificial Intelligence; AUC, Area Under the Curve; CE-MDR, Conformité Européenne—Medical Device Regulation (EU 2017/745); FDA, U.S. Food and Drug Administration.

A four-stage clinical translation roadmap—from preclinical validation to multicenter trials, regulatory approval, and clinical promotion—can guide real-world adoption (Figure 3). 

Looking ahead, AI does not replace clinical judgment; it amplifies precision and timeliness when embedded in co-designed workflows with explicit decision thresholds, utility metrics, and clear governance. If these conditions are met, the convergence of AI-ECG, on-device wearables, ML-guided mapping, multiparametric CIED analytics, DL-enhanced imaging, GNNs, and DT can yield a truly data-driven, patient-specific electrophysiology, where therapeutic choices are simulated before execution, risk windows are identified before clinical destabilization, and uncertainty is surfaced and managed, delivering concrete, measurable benefits for patients.

## Figures and Tables

**Table 1:** Clinically approved applications. Clinically approved solutions include Apple Watch, AliveCor KardiaMobile, Withings ScanWatch, Fitbit, Samsung and FibriCheck and clinical patches (iRhythm, Rooti Rx). AF, Atrial Fibrillation; AFL, Atrial Flutter; AUC, Area Under the Curve; CE, CE mark (Conformité Européenne); ECG, Electrocardiogram; FDA, U.S. Food and Drug Administration; iECG, Single-Lead Integrated ECG; IHRN, Irregular Heart Rhythm Notification; PPG, Photoplethysmography; PPV, Positive Predictive Value; vs., Versus.

Device	Core Performance Indicators	Regulatory Status
Apple Watch (iECG + IHRN PPG)	AF detection sensitivity 94.8%; specificity 95%; AUC 0.96 [32]	FDA/CE
AliveCor KardiaMobile (iECG)	AF detection sensitivity 100%; specificity 97% [33]	
Withings ScanWatch (iECG)	AF detection sensitivity 78%, specificity 80% [34]	
iRhythm Zio^®^ services (patch for continuous ECG)	Diagnostic yield vs. Holter in AF diagnosis 6.8% vs. 5.4%; median time-to-diagnosis 103 vs. 530 days [35]	
Rooti Rx (patch for continuous ECG)	Diagnostic yield vs. Holter in overall arrhythmia detection 59.5% vs. 19.0% (*p* < 0.001); in AF/AFL 9.5% vs. 3.8% [38]	
Fitbit (IHRN PPG)	AF detection PPV 98.2%; sensitivity 67.6% at episode level; specificity 98.4% [23]	FDA
Samsung Galaxy Watch (iECG + IHRN PPG)	AF detection sensitivity 85%; specificity 75% [31]	
FibriCheck (IHRN PPG)	AF detection sensitivity 96%; specificity 97% [36]	CE

**Table 2:** Core parameters of multiparametric CIED models. This table summarizes the main device-derived inputs used by contemporary multiparametric algorithms embedded in CIED. Each combines daily trends from multiple sensors. AF, Atrial Fibrillation; AT, Atrial Tachyarrhythmia; CIED, Cardiac Implantable Electronic Device; HF, Heart Failure; HFRS, Heart Failure Risk Status; HR, Heart Rate; HRV, Heart-Rate Variability; RR, Respiratory Rate; RSBI, Rapid Shallow-Breathing Index; VP, Ventricular Pacing; VT, Ventricular Tachycardia; VF, Ventricular Fibrillation.

Algorithm	Core Inputs (CIED-Derived)	Weights Availability
HeartLogic (Boston Scientific)	Activity Night HR Respiration (RR/RSBI) Heart sounds (S3/S1) Thoracic impedance	Composite index; weights not public [51,52]
TriageHF/HFRS (Medtronic)	% VP Activity AT/AF burden HRV Night HR OptiVol (intrathoracic impedance) Treated VT/VF/shocks Ventricular rate during AT/AF	Rule/Bayesian tiers; no numeric weights [53]
HeartInsight (Biotronik)	24 h HR Activity AT/AF burden HFRS HRV Night HR Thoracic impedance Ventricular extrasystoles	Single score; no fixed % weights [54,55]

**Table 3:** Supervised machine-learning methods in cardiac electrophysiology. Summary of common algorithms, core method, and representative clinical applications in EP. In supervised learning a model is trained on human-labeled inputs and output datasets, in order to identify a set of patterns that can be used for mapping the features of interest on correct outcomes. For instance, this allows for addressing clinical questions. AF, Atrial Fibrillation; CPU, Central Processing Unit; ECG, Electrocardiogram; EGM, Electrogram; GPU, Graphics Processing Unit.

Algorithm	Description	Applicable Scenarios	Data Requirements	Computational Resources
Least-squares regression	Models the relationship between a continuous outcome and inputs by minimizing the sum of squared residuals.	o Risk scoring and regression models o Survival/time-to-event analysis o Simple interpretable baselines	o n ≳ 200–500 o High-quality labels o Low–moderate feature count	CPU only
Support vector machine	Classifies responses by learning a separating hyperplane between groups.	o Small-sample (<1000) ECG datasets o Arrhythmia classification o Prognostic or risk stratification	o n < 1000 o Curated labels o Scaled features	CPU/GPU optional
Neural networks	Learn patterns in data via layered processing of inputs to produce predicted outputs.	o Large-sample ECG or EGM analysis (≥5000) o Automated diagnosis o Complex pattern recognition	o n ≥ 5000–10,000 o Consistent labeling o Benefits from augmentation	GPU required
Random forest	Builds an ensemble of decision trees from bootstrapped samples to predict outcomes.	o Multi-feature registry data (>5000) o Outcome prediction o Feature importance ranking	o n > 5000 o Handles noisy data o Limited preprocessing	CPU sufficient

**Table 4:** Unsupervised machine-learning methods in cardiac electrophysiology. Summary of common algorithms, core method, and representative clinical applications in EP. In unsupervised learning no predefined labels or ground-truth outputs are available. The algorithm autonomously identifies the set of patterns by analyzing latent structures, relationships, and clusters within the data. CPU, Central Processing Unit; DBSCAN, Density-Based Spatial Clustering of Applications with Noise; ECG, Electrocardiogram; EGM, Electrogram; GPU, Graphics Processing Unit; PCA, Principal Component Analysis; T-SNE, T-Distributed Stochastic Neighbor Embedding; UMAP, Uniform Manifold Approximation and Projection.

Algorithm	Description	Applicable Scenarios	Data Requirements	Computational Resources
Clustering (k-means, DBSCAN)	Automatically groups data into homogeneous clusters based on similarity.	o Patient phenotyping o Signal or conduction pattern categorization o Disease subtype identification	o n = hundreds–tens of thousands o No labels o Standardized features preferred	CPU only
Dimensionality reduction (PCA, t-SNE, UMAP)	Reduces input dimensionality and derives principal components or low-dimensional embeddings that preserve relationships among data points.	o Feature selection and visualization o Mapping of electroanatomical data o Exploratory pattern analysis	o n ≥ 500 o No labels o Normalization improves PCA; t-SNA/UMAP for nonlinear patterns	CPU for PCA; GPU optional for t-SNE/UMAP
Association Rule Learning	Identifies frequent co-occurrences/correlations among events or features in clinical data	o Discovery of relations between procedural parameters and outcomes o Risk factor combination identification	o Large categorical datasets o Event coding consistency matters	CPU only
Unsupervised deep learning	Automatically extracts salient features from complex data without labels.	o ECG/EGM signal denoising o Learning compact representations of complex clinical data o Anomaly detection or pre-training for supervised models	o n ≥ several thousand o Unlabeled signals o Broad physiologic variability improves robustness	GPU required

**Table 5:** Emerging AI technologies in cardiac electrophysiology. Compact overview of core principles, data inputs, applications, and clinical impact for key technologies. 3D, Three Dimensional; AF, Atrial Fibrillation; CMR, Cardiovascular Magnetic Resonance; CNN, Convolutional Neural Network; CRT, Cardiac Resynchronization Therapy; CT, Computed Tomography; CV, Conduction Velocity; EAM, Electroanatomical Mapping; EAT, Epicardial Adipose Tissue; ECGI, Electrocardiographic Imaging; EGM, Electrogram; EP, Electrophysiology; GCNN, Graph Convolutional Neural Network; ICE, Intracardiac Echocardiography; LA, Left Atrium; LGE, Late Gadolinium Enhancement; PPG, Photoplethysmography; VT, Ventricular Tachycardia.

Technology	Principle	Inputs	Applications in EP	Potential Clinical Impact
Digital twin	Patient-specific electromechanical models	o ECG o EGM o Echocardiography. o CT o LGE-CMR	o Virtual ablation and minimal target selection o Virtual pacing for CRT	o Personalized strategies o Shorter/safer procedures o Improved CRT candidate selection
Physics-informed neural networks	Neural networks constrained by physical laws	o Sparse/noisy activation maps o 3D geometries o ECGi	o Reconstruction of activation/potentials o Estimation electrophysiological parameters o Inverse ECGi	o More robust maps with fewer measurements o Faster, more reliable mapping.
Deep learning for imaging	CNN/U-Net for segmentation, tissue quantification, and multimodal prediction	o Echocardiography o CT o CMR-LGE o ICE	o LA/myocardial segmentation o Fibrosis/EAT quantification o AF/CRT outcome prediction o ICE view guidance	o Standardized, accelerated workflows o More precise planning o Reduced operator variability.
Graph convolutional neural network	Graph learning over irregular point sets with graph convolutions.	o ECG o EGM o EAM o LGE-CMR o ECGi	o Critical isthmus identification o 3D activation from few points o Next best site o Fewer electrodes for ECGi.	o Shorter procedures with maintained accuracy o More consistent ablation targets
Advanced wearables and on-device AI	Real-time, on-device signal analysis	o ECG o PPG o Multiparameter sensors o Longitudinal data.	o AF screening o Post-cryptogenic-stroke surveillance o Proactive telemonitoring.	o Population-scale screening o Timelier management o Integration into care pathways.

**Table 6:** Advantages and limitations of AI in electrophysiology. Summary of key clinical/organizational benefits and methodological/implementation challenges across screening, EP lab, and follow-up. AF, Atrial Fibrillation; AI-ECG, AI Applied To ECG; CHEERS, Consolidated Health Economic Evaluation Reporting Standards; CRT, Cardiac Resynchronization Therapy; EGM, Intracardiac Electrogram; EP, Electrophysiology; EHR, Electronic Health Record; HF, Heart Failure; Mlops, Machine Learning Operations; OOD, Out Of Distribution; VT, Ventricular Tachycardia; XAI, Explainable AI.

Dimension	Key Benefits	Key Concerns
Advantages		
Scalability	Population-level screening and risk stratification with low marginal cost per evaluation	Risk of under-representation; Excessive alerts without established escalation protocols.
Standardization	More consistent EGM interpretation and substrate tagging; reduced inter-operator variability; reproducible mapping/lesion selection	Over-reliance on algorithmic labels; need for external validation and periodic recalibration across centers/devices.
Timeliness	Earlier detection of HF decompensation and arrhythmic instability via remote multiparametric analytics; faster, safer workflows in-lab.	False positives/negatives can consume resources or delay care; requires monitored thresholds and clinician oversight.
Personalization	Patient-specific planning via digital twins; image- and signal-informed models tailor ablation/CRT and follow-up.	Data quality and model calibration are critical; individual simulations require robust workflow and governance.
System-wide impact	Equitable screening access, reproducible lab decisions, proactive remote care, and improved resource allocation	Benefits are conditional on operational readiness, resourcing, and pathway integration.
Limitations		
Generalizability	Multicenter datasets and external testing improve portability of models across sites and hardware.	Dataset shift, selection bias, and noisy labels degrade performance; demands continuous performance monitoring.
Interpretability	Task-relevant, physiology-aligned explanations increase clinician trust and actionability.	Superficial saliency maps can mislead; require stability testing and uncertainty quantification.
Safety	Prospective evaluation, post-market monitoring (MLOps), clear human-in-the-loop controls for uncertainty/OOD.	Governance overhead with the need for audit trails, rigorous versioning, and controls to suspend or revert models.
Organizational readiness	Seamless EHR/mapping integration; defined roles; trained teams; medico-legal clarity.	Integration costs/time; unclear accountability can stall or negate value.
Economic value	Potential cost offsets via fewer rehospitalizations, shorter procedures/fluoroscopy, targeted follow-up.	Must be proven in pragmatic trials with CHEERS-quality economic reporting; hidden costs of maintenance and updates.

## Data Availability

No new data were created for this review article.

## Abbreviations

The following abbreviations are used in this manuscript:

3D	Three Dimensional
AF	Atrial Fibrillation
AFib	Atrial Fibrillation
AIx	Ablation Index
AI	Artificial Intelligence
AI-ECG	Artificial-Intelligence Electrocardiography
APD	Action Potential Duration
ARL	Association Rule Learning
BPV	Blood-Pressure Variability
CABANA	Catheter ABlation vs. ANtiarrhythmic Drug Therapy for Atrial Fibrillation (trial)
CDSS	Clinical Decision Support Systems
CE	Conformité Européenne
CHEERS	Consolidated Health Economic Evaluation Reporting Standards
CIED	Cardiac Implantable Electronic Device
CMR	Cardiovascular Magnetic Resonance
CNN	Convolutional Neural Network
CRT	Cardiac Resynchronization Therapy
CT	Computed Tomography
CV	Conduction Velocity
CVAE	Conditional Variational Autoencoder
DBSCAN	Density-Based Spatial Clustering of Applications with Noise
DL	Deep Learning
DPIA	Data Protection Impact Assessment
DT	Digital Twin
EAM	Electroanatomical Mapping
EAT	Epicardial Adipose Tissue
ECG	Electrocardiogram
ECGi	Electrocardiographic Imaging
EGM	Intracardiac Electrogram
edge-AI	On-device/edge Artificial Intelligence
EHR	Electronic Health Record
EU	European Union
FDA	Food and Drug Administration
FU	Follow Up
GCNN	Graph Convolutional Neural Network
GDPR	General Data Protection Regulation
GMLP	Good Machine Learning Practice
HF	Heart Failure
HIPAA	Health Insurance Portability and Accountability Act
HRV	Heart-Rate Variability
ICD	Implantable Cardioverter-Defibrillator
ICE	Intracardiac Echocardiography
inFAT	CT-derived intramyocardial/epicardial fat marker
LA	Left Atrium
LGE-CMR	Late Gadolinium Enhancement Cardiac Magnetic Resonance
LSI	Lesion Size Index
LSR	Least-Squares Regression
MDR	Medical Device Regulation
MLOps	Machine Learning Operations
NN	Neural Network(s)
OOD	Out-Of-Distribution
PCA	Principal Component Analysis
PCCP	Predetermined Change Control Plan
PINN	Physics-Informed Neural Network
PMA	Premarket Approval
PPG	Photoplethysmography
PTB-XL	Public ECG dataset “PTB-XL”
PTB-XL+	Extended release of PTB-XL
PVI	Pulmonary Vein Isolation
RA	Right Atrium
RF	Radiofrequency
RVI	Reentry Vulnerability Index
SaMD	Software as a Medical Device
SSI	Systolic Stretch Index
t-SNE	t-Distributed Stochastic Neighbor Embedding
TinyML	Tiny/embedded Machine Learning
U-Net	U-Net convolutional architecture
UMAP	Uniform Manifold Approximation and Projection
VF	Ventricular Fibrillation
VT	Ventricular Tachycardia
XAI	Explainable Artificial Intelligence
